# Genome Wide Identification and Expression Profiling of Ethylene Receptor Genes during Soybean Nodulation

**DOI:** 10.3389/fpls.2017.00859

**Published:** 2017-06-12

**Authors:** Youning Wang, Jinhong Yuan, Wei Yang, Lin Zhu, Chao Su, Xiaodi Wang, Haiyan Wu, Zhengxi Sun, Xia Li

**Affiliations:** ^1^State Key Laboratory of Agricultural Microbiology, College of Plant Science and Technology, Huazhong Agricultural UniversityWuhan, China; ^2^State Key Laboratory of Plant Cell and Chromosome Engineering, Institute of Genetics and Developmental Biology – Chinese Academy of SciencesBeijing, China; ^3^University of Chinese Academy of SciencesBeijing, China

**Keywords:** soybean, *Glycine max*, ethylene, ethylene receptors, nodulation

## Abstract

It has long been known that the gaseous plant hormone ethylene plays a key role in nodulation in legumes. The perception of ethylene by a family of five membrane-localized receptors is necessary to trigger the ethylene signaling pathway, which regulates various biological responses in *Arabidopsis*. However, a systematic analysis of the ethylene receptors in leguminous plants and their roles in nodule development is lacking. In this study, we performed a characterization of ethylene receptor genes based on the latest *Glycine max* genome sequence and a public microarray database. Eleven ethylene receptor family genes were identified in soybean through homology searches, and they were divided into two subgroups. Exon–intron analysis showed that the gene structures are highly conserved within each group. Further analysis of their expression patterns showed that these ethylene receptor genes are differentially expressed in various soybean tissues and organs, including functional nodules. Notably, the ethylene receptor genes showed different responses to rhizobial infection and Nod factors, suggesting a possible role for ethylene receptors and ethylene signaling in rhizobia–host cell interactions and nodulation in soybean. Together, these data indicate the functional divergence of ethylene receptor genes in soybean, and that some of these receptors mediate nodulation, including rhizobial infection, nodule development, and nodule functionality. These findings provide a foundation for further elucidation of the molecular mechanism by which the ethylene signaling pathway regulates nodulation in soybean, as well as other legumes.

## Introduction

Symbiotic nitrogen fixation (SNF) plays critical roles in legume development and yield. SNF efficiency is determined by rhizobial infection, nodule development, and mature nodule functionality. Nodulation is initiated by flavonoids secreted from legume roots in response to nitrogen-limited conditions, which stimulate rhizobia to synthesize and secrete lipooligosaccharides called Nod factors (NFs). NFs are perceived by NF receptors, which activate signaling cascades that promote root hair deformation, the formation and growth of infection threads, cortical and pericycle cell division, and nodule development (Stacey et al., 2006; Oldroyd and Downie, 2008; Gresshoff et al., 2009; Ferguson et al., 2013). Nodulation is thus a complex process that is precisely and dynamically regulated by both internal and external cues (reviewed in Ferguson et al., 2010).

The gaseous hormone ethylene plays multiple roles in plant development, including seed dormancy, fruit ripening, flower and leaf senescence, and plant responses to environmental cues (Abeles et al., 1992; Johnson and Ecker, 1998; Bleecker and Kende, 2000). In *Arabidopsis*, the core ethylene signal transduction pathway has been well-characterized (Kieber et al., 1993; Chen et al., 2002; Ju et al., 2012; Cho and Yoo, 2015). In *Arabidopsis*, ethylene is perceived by Ethylene Receptor 1 (ETR1)/ETR2 (Chen et al., 2002), Ethylene Response Sensor (ERS) 1/ERS2, and Ethylene Insensitive (EIN) 4. These proteins comprise a family of endoplasmic reticulum (ER) membrane-associated proteins that negatively regulate ethylene signaling (Bleecker and Kende, 2000; Chang and Stadler, 2001). Upon binding ethylene, these receptors are inactivated, allowing them to bind Ethylene Insensitive 2 (EIN2) more efficiently (Cho and Yoo, 2015) and block its phosphorylation by Constitutive Triple Response 1 (Kieber et al., 1993). The C-terminus of EIN2 is then cleaved, inducing a conformational change in the protein that enables it to move to the nucleus and enhance the transcriptional activity of the transcription factors EIN3 and EIN3-Like 1 (EIL1). EIN3 and EIL1 activate ethylene-responsive genes such as *Ethylene Response Factor 1* (*ERF1*) to switch on ethylene signaling (Ju et al., 2012). Clearly, the perception of ethylene by specific receptor proteins is essential for ethylene action (Burg and Burg, 1967). Over the past several decades, extensive effort has been made to elucidate how these receptors perceive ethylene to activate ethylene signaling. Based on a structural analysis, the receptors have been divided into two subgroups. The type I subfamily includes ETR1 and ERS1, which consist of two critical domains, an N-terminal ethylene-binding domain (the sensor domain) and a well-conserved C-terminal histidine (His) kinase domain (Guo and Ecker, 2004). The type II subfamily consists of ETR2, ERS2, and EIN4, which contain an N-terminal ethylene-binding domain and a degenerate His kinase domain lacking one or more elements necessary for kinase catalytic activity (Guo and Ecker, 2004). In addition, ETR1, ETR2, and EIN4 have a C-terminal receiver domain of unknown function (Guo and Ecker, 2004). Despite their structural differences, all of these receptors are involved in ethylene perception and redundantly regulate ethylene-mediated biological processes affecting plant development and interactions with environment (Schaller and Bleecker, 1995; Hua and Meyerowitz, 1998; Guo and Ecker, 2004).

The negative effect of ethylene on legume nodulation was documented 40 years ago in experiments using exogenously applied ethylene or an inhibitor of ethylene biosynthesis (aminoethoxyvinylglycine) (Grobbelaar et al., 1971; Goodlass and Smith, 1979; Guinel and LaRue, 1992; Lee and Larue, 1992; Penmetsa and Cook, 1997; Nukui et al., 2000; Lohar et al., 2009). In recent years, compelling experimental evidence has demonstrated the crucial role of ethylene and ethylene signaling in nodulation in legumes. Ethylene is induced by NFs, and fluctuations in ethylene levels have been detected during nodulation (Gamalero and Glick, 2015). Notably, a recent study showed that ethylene positively or negatively regulates early (i.e., 1 h after inoculation) and late (6 h after inoculation) rhizobial infection *via* NF-independent and -dependent pathways, respectively (Larrainzar et al., 2015). The authors further proposed that these regulatory pathways are responsible for the different effects of ethylene on biological processes: the former in defense, and the latter in the initiation of nodulation (Larrainzar et al., 2015). Based on these results, it appears that ethylene is a master regulator of nodulation that affects multiple hormonal signaling pathways to regulate every step of the process, including rhizobial infection, nodule organogenesis, and nodule senescence (Guinel, 2016). Despite great progress in understanding the involvement of ethylene in nodulation, genetic evidence for the role of ethylene signaling comes mainly from functional analyses of loss-of-function mutants of *Arabidopsis* EIN2 orthologs. The ‘*sickle*’ mutant of *Medicago truncatula* which carries a loss-of-function mutation in *MtEIN2*, is insensitive to ethylene and forms 10–30 times more nodules than wild-type plants (Penmetsa and Cook, 1997; Penmetsa et al., 2008). The mutation of two *Lotus EIN2* genes was also shown to cause hypernodulation in *Lotus japonicus* (Miyata et al., 2013), highlighting the conserved role of the ethylene signaling pathway in legume nodulation. The fact that the transgenic *Lotus japonicus* harboring the mutated *Arabidopsis ETR1 or Cm-ERS1/H70A* reduced ethylene sensitivity and enhanced nodulation (Nukui et al., 2004; Lohar et al., 2009) supports the notion that canonical ethylene perception and its signaling transduction show a significant role during nodulation in legumes. However, the role of ethylene in soybean nodulation remains controversial. Several studies have shown that neither an increase in ethylene production nor treatment with aminoethoxyvinylglycine affects nodule formation (Lee and Larue, 1992; Hunter, 1993; Suganuma et al., 1995); however, one study showed increased nodule numbers in soybean plants treated with ethylene inhibitors (Caba et al., 1998). It was suggested that this controversial result might be due to the experimental methodology (Schmidt et al., 1999). However, a phenotypic analysis of an ethylene-insensitive mutant, *etr1-1*, supports the idea that ethylene is less significant in nodule development in soybean compared to other plants because the nodule number in *etr1-1* was comparable to that in wild-type (Schmidt et al., 1999). Thus, the roles of ethylene in rhizobia–soybean interactions and nodule development in soybean are unclear. Moreover, the functions of most genes related to ethylene perception and signal transduction are unknown.

The availability of a transcriptome database and the recent sequencing of the soybean genome provided us with tools to examine which genes are involved in nodulation, and they provided us with clues about whether known ethylene-related genes mediate nodulation in soybean. To gain insight into the roles of ethylene signaling in soybean, especially in nodulation, we performed a genome wide search for soybean homologs of the *Arabidopsis* ethylene receptor genes *ETR1, ETR2, ERS1, ERS2*, and *EIN4*. Detailed analyses of the structures, phylogeny, conserved domains, and expression profiles of these genes were performed. In addition, the expression patterns of the genes in response to rhizobial inoculation, NFs and in functional nodules were analyzed using quantitative real-time reverse transcription-polymerase chain reaction (qRT-PCR). Through these analyses, we uncovered structural and functional divergence among soybean ethylene receptor genes and proteins. Our results provide a framework for the further functional characterization of ethylene receptor family genes in soybean.

## Materials and Methods

### Phylogenetic Tree and Gene Structure Analysis

We obtained the sequences of the identified ethylene receptor genes from a published database (Phytozome^1^), including genomic DNA sequences, coding sequences, and amino acid sequences. A phylogenetic tree was constructed based on the amino acid sequences of all putative ethylene receptor genes using Clustal X 1.83 (Thompson et al., 1997) and MEGA6.0 (Tamura et al., 2011). The structures of the ethylene receptor genes were determined using the Gene Structure Display Server (GSDS) website^2^.

### *Cis*-Element Analysis

1.5 kb sequences upstream of all the ethylene receptor genes were downloaded from *Glycine max* database. The regulatory *cis*-elements were then analyzed using website PlantCARE^3^.

### Expression Data Collection and Heatmap Construction

Expression data for the ethylene receptor genes were collected from SoyBase^4^ (Severin et al., 2010) and the eFP Browser^5^ (Libault et al., 2010). Heatmaps of the ethylene receptor genes were constructed using Heatmap Illustrator v1.0 (Deng et al., 2014).

### Plant Materials and Growth Conditions

Soybean (*G. max* [L.] Merrill) cv. Williams 82 plants were used in this study. To analyze gene expression in response to rhizobial inoculation, soybean plants were grown in vermiculite irrigated with a nitrogen-deficient solution in a growth room (16 h of light/8 h of dark; 25°C) (Wang et al., 2009). Ten-day-old plants were inoculated with *Bradyrhizobium japonicum* strain USDA110 (OD_600_ = 0.08; 30 mL/plant) in the same nitrogen-deficient solution, and roots were collected at specific time points after rhizobial inoculation. To examine the early root response to rhizobial infection, roots were harvested at 0, 1, 3, 6, 12, and 24 h after rhizobial inoculation. To examine the expression pattern of ethylene receptor genes in different tissues, leaves, roots, and nodules were collected at 28 days after rhizobial inoculation. The method of NF application studies was used as described by Wang et al. (2014, 2015). Root samples were collected and used to analyze the expression of ethylene receptor genes at 24 h after NF treatment.

### RNA Extraction and qRT-PCR

To estimate the ethylene receptor gene expression levels, total RNA was extracted from different tissues using Trizol reagent (Tiangen Biotech [Beijing] Co. Ltd, Beijing, China). Aliquots (2 μg) of total RNA were treated with DNase I (Invitrogen, Carlsbad, CA, United States) and used to synthesize first-strand cDNA with a FastQuant RT Kit (Tiangen Biotech [Beijing] Co. Ltd). qRT-PCR was performed using SuperReal PreMix Plus (SYBR Green; Tiangen Biotech [Beijing], Co., Ltd) on an ABI 7500 Real-Time PCR System (Invitrogen). *GmELF1b* (*Eukaryotic elongation factor 1-beta*) was used as an internal control (Jian et al., 2008). The primers used in this study are listed in Supplementary Table S1.

### Statistical Analysis

The expression data were analyzed by Student’s *t*-test or one-way analysis of variance using SigmaPlot 10.0 or GraphPad Prism 5 software. Different letters indicate a significant difference in the relative gene expression (*P* < 0.05). Moreover, statistically significant differences were indicated as follows: ^∗^*P* < 0.05; ^∗∗^*P* < 0.01; ^∗∗∗^*P* < 0.001; ns, not significant, *P >* 0.05.

## Results

### Genome Wide Identification of Ethylene Receptor Genes in Soybean

Based on the data collected from the Phytozome website, five ethylene receptor genes, *AtETR1, AtERS1, AtETR2, AtEIN4*, and *AtERS2*, were used as queries against the *G. max* genome in the Plant Genome Duplication Database (Lee et al., 2012). In total, 11 homologous genes were found in the soybean genome. Except that, there has 4 homologous genes in rice and six homologous genes in *Medicago*. Basic information about homologous genes in soybean is provided in Supplementary Table S2. The deduced proteins encoded by these putative ethylene receptor genes contain 636–768 amino acid residues, and their molecular masses range from 63.6 to 76.8 kDa, similar to the ethylene receptors of *Arabidopsis*. In addition, the isoelectric points of the proteins encoded by these soybean ethylene receptor genes range from 6.19 to 8.17.

### Phylogenetic and Structure Analyses of the Soybean Ethylene Receptor Genes

**FIGURE 1:**
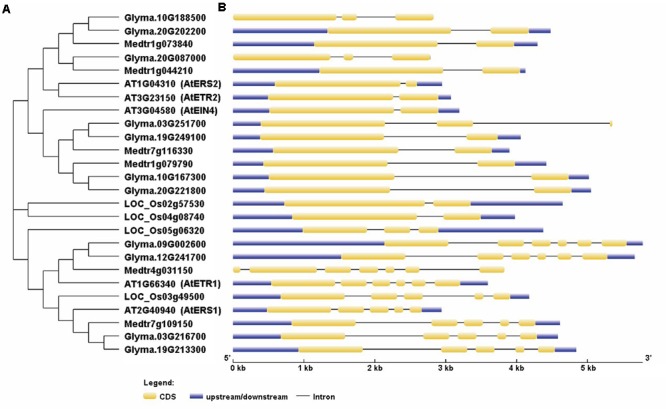
**Phylogenetic relationships of the ethylene receptor family proteins and their gene structures.** The phylogenetic tree **(A)** was constructed using MEGA6.0, while the structures of the ethylene receptor genes **(B)** were created using the GSDS website.

**FIGURE 2:**
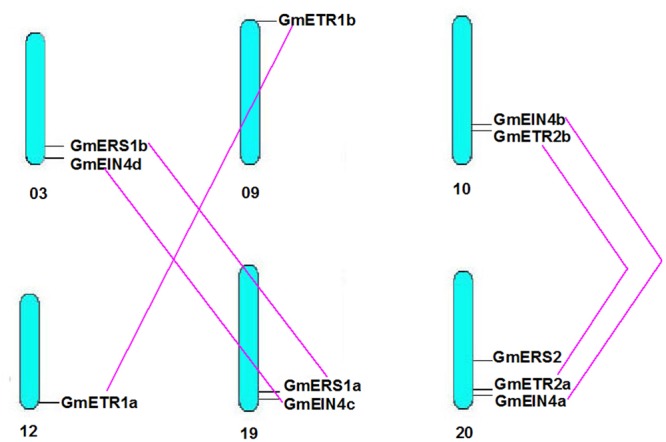
**Chromosomal distribution of the identified ethylene receptor genes.** Based on soybean genome annotation v1.1, the chromosomal locations of the homologous ethylene receptor genes in soybean were analyzed and marked on the corresponding chromosomes from top to bottom. Duplicated genes are marked with a purple line.

Further analysis showed that the 11 homologs are located on six different chromosomes three on chromosome 20, two on chromosomes 3, 10, and 19; and one on chromosomes 9 and 12 (Supplementary Table S2). To assess the phylogenetic relationships among the soybean and *Arabidopsis* receptor proteins, we constructed a phylogenetic tree with the protein sequences of the ethylene receptors by using the neighbor-joining method in MEGA6.0 (Tamura et al., 2011). To see whether other leguminous plants contain all the ethylene receptor genes, we also included the ethylene receptor proteins in *Medicago truncatula*. Our results indicate that, in soybean, four of the genes are homologs of *AtEIN4*, two are homologs of *AtETR1* and *AtERS1*, respectively, and three are homologs of *AtERS2* and *AtETR2* (**Figure 1A**). Notably, Glyma.20g08700 has been designated as *GmERS2* in PGDD. The gene names and locus IDs of these receptor genes are listed in Supplementary Table S2. In *Medicago*, totally six ethylene receptor genes were identified: one homolog for *MtETR1* and *MtERS1*, respectively; two homologs for *MtEIN4* and two potential homologs for *MtETR2* and *MtERS2*,

We next analyzed the structures of the ethylene receptor genes using GSDS 2.0^6^, and the mRNA and genomic DNA sequences were downloaded from the Phytozome database. As shown in **Figure 1**, the gene structures of the homologs of *AtERS1* and *AtETR1* are highly conserved and have the same exon–intron pattern. However, the four homologs of *AtEIN4* exhibited two structural patterns. The structures of *Glyma.20g221800* (*GmEIN4a*), *Glyma.10g167300* (*GmEIN4b*), and *Glyma.19g249100* (*GmEIN4c*) were similar, whereas the structure of *Glyma.03g251700* (*GmEIN4d*) was different, with three exons and two introns (**Figure 1B**). In terms of the *AtETR2* and *AtERS2* homologs, *Glyma.10g188500* (*GmETR2b*) and *Glyma.20g087000* (*GmERS2*) each have three exons and two introns, in contrast to *AtETR2* and *AtERS2*, which contain one intron and two exons each. A duplication analysis conducted using EnsemblPlants^7^ identified five pairs of duplicates. As shown in **Figure 2**, the duplicated pairs are *GmETR1a-GmETR1b, GmERS1a-GmERS1b, GmETR2a-GmETR2b, GmEIN4a-GmEIN4b*, and *GmEIN4c-GmEIN4d*.

### Protein Structure Analysis of the Ethylene Receptors in Soybean

**FIGURE 3:**
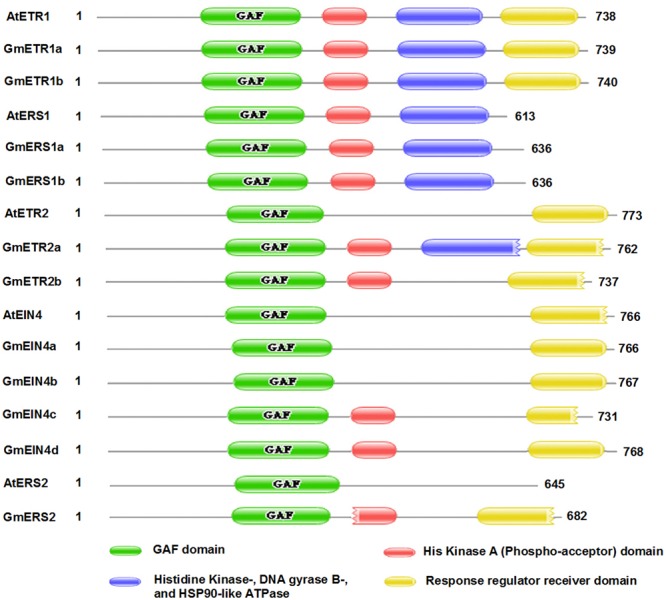
**The conserved domains of the homologous ethylene receptor proteins in soybean.** The conserved domains of the homologous proteins were analyzed using the PFAM website (http://pfam.xfam.org/).

It has been proposed that ethylene receptor family proteins are highly conserved in plants, and that they are typical ER membrane-associated proteins sharing similarity with bacterial two-component regulators (Chang and Stadler, 2001; Guo and Ecker, 2004). To obtain detailed information about the structures of these ethylene receptor proteins in soybean, the deduced amino acid sequences collected from the Phytozome database were aligned, and the proteins structures were analyzed using PFAM^8^. As shown in **Figure 3**, the soybean ethylene receptor proteins all contain one GAF domain (green box), just like in *Arabidopsis*; by contrast, the protein domains [e.g., His kinase A (phospho-acceptor) domain (red box), His kinase domain (blue box), and response regulator receiver domain] in the soybean receptors are variable. Notably, the protein structures of GmETR1 and GmERS1 are identical to those of AtETR1 and AtERS1. However, the predicted proteins GmETR2, GmEIN4, and GmERS2 show some variation compared with their *Arabidopsis* homologs. For example, GmETR2, GmEIN4c/d, and GmERS2 contain a His kinase A domain, which does not appear in AtETR2, AtEIN4, and AtERS2 (**Figure 3**). In addition, GmETR2a contains a His kinase domain. It was also found that the receiver domain at the C-terminus shows divergence among most subfamily members, except for ETR1 (**Figure 3**). AtETR2 has a complete receiver domain (yellow box), while GmETR2a and GmETR2b appear to have a degenerate domain lacking one or more elements (**Figure 3** and Supplementary Figure S1). As for the structures of the AtEIN4 homologs, only GmEIN4c was found to have a degenerate domain similar to that of AtEIN4; in contrast, the other three proteins have a complete receiver domain (**Figure 3**). The conservation of these receptors between *Arabidopsis* and soybean indicates that these proteins likely have conserved functions, while the differences in domains between these species suggest that the proteins have divergent functions in soybean.

**FIGURE 4:**
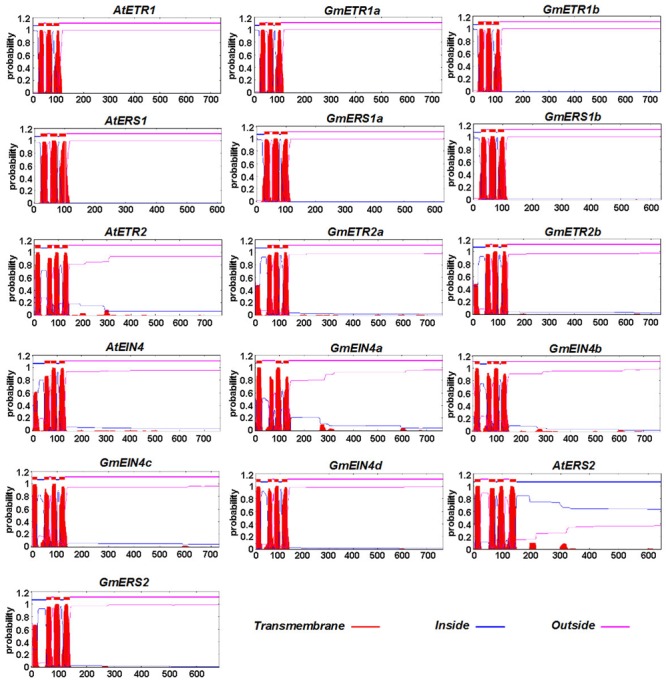
**The predicted protein profiles of the soybean ethylene receptors.** The transmembrane helices of the ethylene receptor proteins were predicted using TMHMM2 (http://www.cbs.dtu.dk/services/TMHMM-2.0/). The transmembrane domains of the predicted proteins are shown by red peaks.

The transmembrane architecture of the ethylene receptor proteins was also predicted using TMHHM 2.0^9^. As shown in **Figure 4**, these ethylene receptors contain several transmembrane domains, although the number of transmembrane domains varies by receptor. Like AtETR1 and AtERS1, GmETR1a/b and GmERS1a/b have three conserved transmembrane domains at their N-terminus. However, the number of typical transmembrane domains in GmETR2 and GmERS2 is reduced compared with AtETR2 and AtERS2, while the number of transmembrane domains in GmEIN4b, GmEIN4c, and GmEIN4d appears to be increased compared with their homologs (**Figure 4**).

### *Cis*-Elements in the Promoters of the Ethylene Receptor Genes in Soybean

**FIGURE 5:**
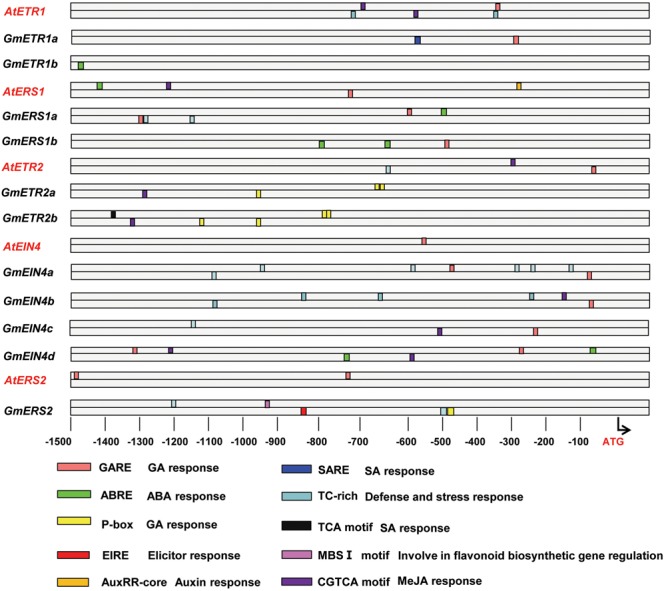
**Promoter analysis of the homologous ethylene receptor genes.** The promoters of the genes (1,500 bp) were analyzed online (http://bioinformatics.psb.ugent.be/webtools/plantcare/html/) to detect *cis*-elements using PLACE software (http://www.dna.affrc.go.jp/PLACE/).

In order to aid in understanding the putative functions of these ethylene receptor genes in soybean, we further performed the promoter analysis to identify the regulatory *cis*-elements that are involved in various biological processes, in particular plant hormonal response, defense response and nodulation. As shown in **Figure 5**, when compared with the conserved *cis*-elements identified from the promoters of *Arabidopsis* ethylene receptor genes and its’ homologous genes in soybean, we found that they share some *cis*-elements, such as CGTCA motif, which might related with MeJA response; but interestingly, some *cis*-elements (i.e., P-box, TCA motif) only appeared in the promoters of the homolog genes in soybean. For example, the P-box, which was also predicted to be involved in GA response, was only observed in the promoters of *GmETR2a, GmETR2b*, and *GmERS2*. Another regulatory *cis*-element unique to soybean ethylene receptors is TC-rich repeat element, which is involved in defense and stress response. TC-rich repeat element occurs in the promoters of ethylene receptors *GmERS1a, GmEIN4a/b/c* and *GmERS2*, although the numbers of the *cis-*element are different; in sharp contrast, only single element was found in the promoters of *Arabidopsis* ethylene receptors *AtETR1* and *AtETR2*. The ABRE elements, which is related to ABA response, were found only in the promoter regions of *GmETR1b, GmERS1a/1b*, and *GmEIN4d* (**Figure 5**). Interestingly, both EIRE element and MBS I element, which were related to Elicitor response and flavonoid biosynthetic gene regulation, respectively, were only observed in the promoter of *GmERS2*. These observations indicate that regulations of the ethylene receptor genes are divergent in soybean and *Arabidopsis* and that the ethylene receptors may modulate different biological processes during plant development and responses to environmental stimuli in soybean and *Arabidopsis*.

### Expression Patterns of Soybean Ethylene Receptor Genes Based on HiSeq Data

**FIGURE 6:**
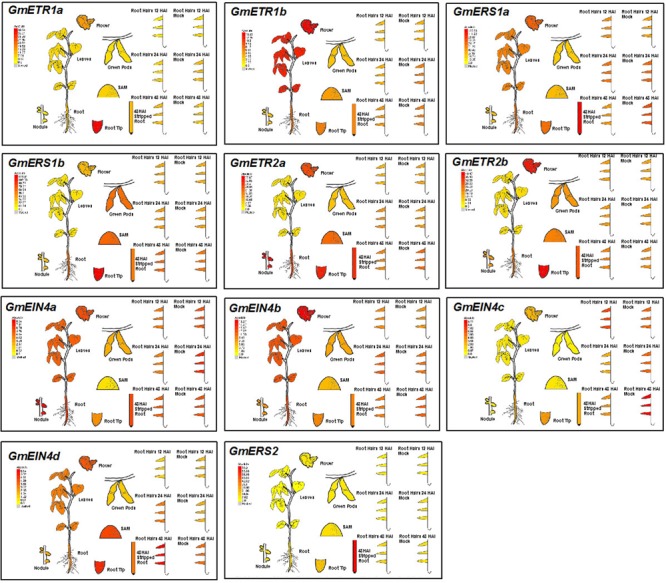
**Expression patterns of the ethylene receptor genes based on Hiseq data.** Based on information from the eFP Browser (http://bar.utoronto.ca/efpsoybean/cgi-bin/efpWeb.cgi), the tissue-specific expression patterns of the ethylene receptor genes and the expression patterns in response to rhizobial inoculation were analyzed.

To investigate the possible roles of the ethylene receptor genes in soybean, we first analyzed the expression patterns of these genes in different tissues and organs including leaves, roots and mature nodules by collecting the HiSeq data from the eFP website (Libault et al., 2010)^10^. As shown in **Figure 6**, these ethylene receptor genes exhibited distinct tissue/organ expression patterns. For example, *GmETR1b, GmERS1a, GmEIN4a, GmEIN4b*, and *GmEIN4d* had relative high expression levels in soybean leaves; *GmETR1b, GmERS1a/b, GmEIN4a, GmEIN4b* were expressed at higher levels in roots. In addition, *GmETR1a* was specifically expressed in root tips, while *GmEIN4c* and *GmERS2* were expressed at relative low levels in all tissues and organs. This observation indicates that the ethylene receptor genes are differentially expressed during soybean growth and development.

Interestingly, *GmETR2a* and *GmEIN4a* were highly expressed in mature nodules (**Figure 6**). Furthermore, several ethylene receptor genes were differentially regulated by rhizobial infection. Among them, the expression of *GmEIN4c* was downregulated by rhizobial inoculation at 24 and 48 h after infection, while *GmERS1a* expression was decreased at 48 h after rhizobial inoculation (**Figure 6**). By contrast, only *GmEIN4d* was induced by rhizobia at 24 h after infection (**Figure 6**). Together, these results suggest that ethylene receptor genes might be involved in rhizobia–soybean interactions and have different functions during nodule development.

### Experimental Validation of Tissue Expression of Soybean Receptor Genes

**FIGURE 7:**
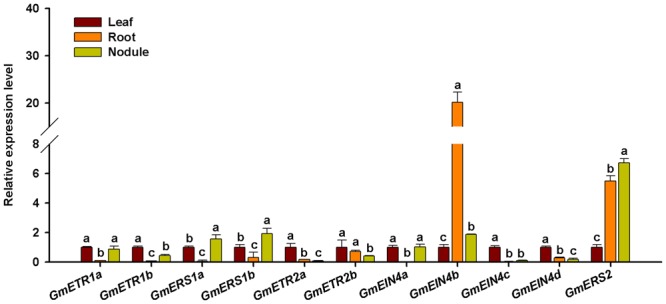
**Expression analysis of the ethylene receptor genes in different tissues of soybean.** The expression patterns of the ethylene receptor genes in leaf, root, and mature nodule of soybean plants at 28 days after rhizobia inoculation were analyzed by qRT-PCR. Expression levels were normalized against the geometric mean of the reference gene *GmELF1b*. Three independent biological repeats were done; the results shown are the averages ± standard deviation. Different letters indicate significant differences in relative gene expression by the Student-Newman-Keuls test (*P* < 0.05).

To validate the tissue specific expression patterns of the ethylene receptor genes, RT-qPCR was used to analyze the expression pattern of those ethylene receptor genes in different tissues of soybean plants at 28 days after rhizobia inoculation. As shown in **Figure 7**, all the ethylene receptor genes were differentially expressed in leaf, root, and nodule. Majority of them were showed higher levels of expression in leaf than in root and nodule. Interestingly, the duplicates of the ethylene receptor genes *GmETR1, GmETR2*, and *GmEIN4* except *GmERS1* exhibited different patterns in the tissues examined. Among 11 ethylene receptor genes, only *GmETR1a* and *GmEIN4a* displayed the same expression pattern. Intriguingly, among the ethylene receptor genes, *GmEIN4b* showed highest expression in root, followed by *GmERS2* in nodule and root.

In addition, association between promoter and gene expression pattern was analyzed based on the digital expression data. The expression data of each gene in eight different tissues of soybean^10^ and 47 tissues of *Arabidopsis* ethylene receptor genes collected from the website^11^ were used to be analyzed (Tamura et al., 2004) (Supplementary Tables S3, S4). As shown in Supplementary Figure S2, the relative correlation coefficient (R^2^) in soybean and *Arabidopsis* were 0.12 and 0.35, respectively. These data indicates that the promoter sequence similarity and expressional pattern of ethylene receptor genes might show positive association. In order to make a further understanding about the ethylene receptor genes, a molecular evolution analysis has been done for pairs of duplicated genes. The coding sequences collected from the Phytozome database were aligned with MEGA6 and parameters between paired genes were estimated with SNAP^12^. dn/ds indicates the ratio of non-synonymous to synonymous substitutions. As shown in Supplementary Table S5, the dn/ds ratio of all paired genes showed lower than one, which suggested that the molecular evolution of these ethylene receptor genes was conservative.

### Expression Validation of Soybean Receptor Genes in Response to Rhizobial Infection and Nod Factor

**FIGURE 8:**
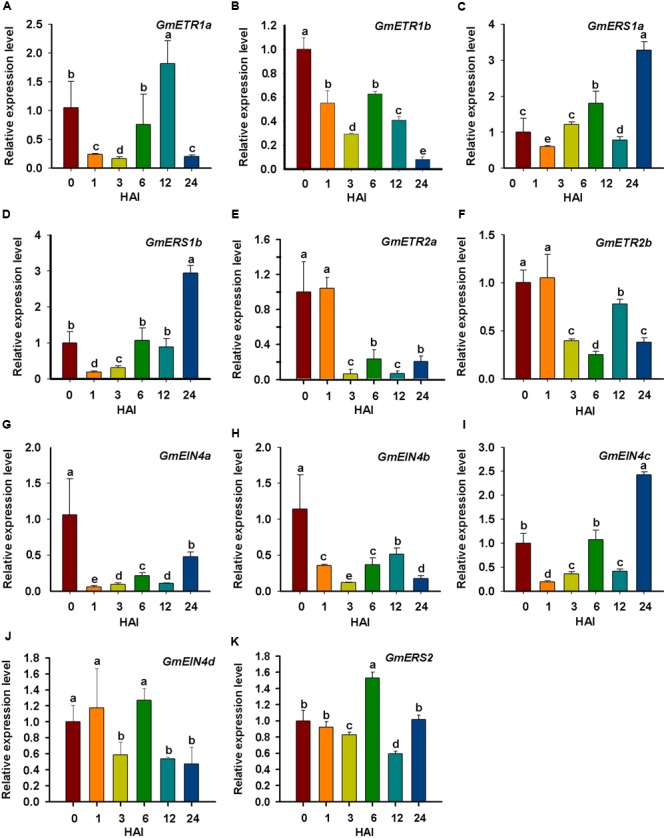
**Expression analysis of the ethylene receptor genes to outline the early response to rhizobial inoculation. (A–K)** The relative expression level of ethylene receptor genes *GmETR1a, GmETR1b, GmERS1a, GmERS1b, GmETR2a, GmETR2b, GmEIN4a, GmEIN4b, GmEIN4c, GmEIN4d*, and *GmERS2* in response to rhizobial inoculation at different time points (0, 1, 3, 6, 12, and 24 h). The short-term expression patterns of the ethylene receptor genes in response to rhizobial inoculation was analyzed by qRT-qPCR. The expression level of each gene was normalized against the geometric mean of the soybean reference gene *GmELF1b*. Three independent biological repeats were done; the results shown are the averages ± standard deviation. Different letters indicate significant differences by the Student-Newman-Keuls test (*P* < 0.05).

Because we were interested in the roles of ethylene receptor genes in soybean nodulation, we performed qRT-PCR assays to analyze the expression patterns of soybean ethylene receptor genes in response to rhizobial infection and during early process of nodulation. Soybean seedlings were inoculated with rhizobial strain *B. japonicum* USDA110, and root samples were collected at specific time points. The qPCR analysis results detected variable expression of the ethylene receptor genes in response to rhizobia infection (**Figure 8**). Among them, *GmERS1a, GmERS1b*, and *GmEIN4c* were highly induced within 24 h (**Figures 8C,D,I**), while the expression of *GmETR1b, GmETR2a, GmETR2b, GmEIN4a*, and *GmEIN4b* were significantly repressed within 24 h (**Figures 8B,E–H**). The rest of the ethylene receptor genes *GmETR1a, GmEIN4d*, and *GmERS2* were slightly upregulated and then downregulated during the experimental period of time (**Figures 8A,J,K**). These results confirm that majority of soybean ethylene receptor genes are responsive to rhizobial infection.

**FIGURE 9:**
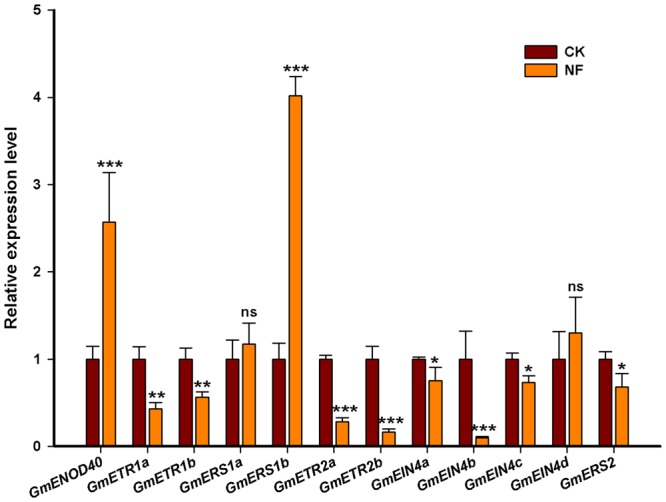
**Expression analysis of the ethylene receptor genes in response to Nod factor (NF).** The expression pattern of the ethylene receptor genes was analyzed by qRT-qPCR in response to NF (10^-8^ M) treatment, where *GmENOD40* was chosen to be a positive control. The expression was normalized against the geometric mean of the reference gene *GmELF1b*. The experiments were done for three independent times. Error bars indicate SD. Statistically significant difference (Student’s *t*-test) are indicated as follows: ns, no significance *P* > 0.05; ^∗^*P* < 0.05; ^∗∗^*P* < 0.01; ^∗∗∗^*P* < 0.001.

To further confirm the responsiveness of the ethylene receptor genes, we also tested whether these genes could be affected by NF. Four-day-old soybean seedlings were treated with NF for 24 h, and root samples were collected for qRT-PCR. As shown in **Figure 9**, compared with the induction of early nodulin gene *GmENOD40*, the transcript levels of *GmETR1a/b, GmETR2a/b, GmEIN4a/b, GmEIN4c*, and *GmERS2* were repressed significantly in response to NF application in soybean roots. However, the expression of *GmERS1a* and *GmEIN4d* were not significantly changed in response to NF treatment compared with the untreated control. Notably, the expression of *GmERS1b* was highly induced by NF and the expression level of *GmERS1b* was increased about fourfold (**Figure 9**). These results suggest that ethylene receptor genes may mediate rhizobia–plant cell interaction and early nodule development in soybean.

## Discussion

Ethylene receptors are central regulators that turn the downstream ethylene signaling transduction pathway on/off. Since ethylene controls various biological processes in different tissues/organs and at different developmental stages, plants have evolved multiple ethylene receptors with divergent gene structures, protein structures, kinase activities, and patterns of transcriptional regulation to precisely and dynamically modulate plant responses to ethylene. Compelling evidence suggests that ethylene receptors regulate plant development and plant responses to environmental stimuli in a complex manner. In this study, we performed a systematic analysis of the ethylene receptors in soybean, including their gene structures, conserved domains, and gene expression patterns in response to rhizobial infection. Our results provide an overview of the main characteristics of these receptors and their potential functions in soybean.

Previous studies have shown that the numbers of ethylene receptors vary in different plant species (Cao et al., 2003; Gallie, 2015b). For example, *Arabidopsis* and rice both have five ethylene receptors, although the types are different (Cao et al., 2003). The number of ethylene receptors in a species represents the level of functional complexity in terms of ethylene perception and cellular responses. Soybean is an allotetraploid plant, and most of its genes have several duplicates (Shoemaker et al., 2006; Schlueter et al., 2007). By searching the soybean genome and gathering data from PGDD, we found 11 ethylene receptors instead of 8 (GmETR1b, GmETR2a, and GmERS2 were missing) used in the previous study (Gallie, 2015a). A phylogenetic analysis showed that soybean has homologs of all of the ethylene receptors in *Arabidopsis*, but that these receptors were not equally duplicated during evolution. Among them, *GmETR1, GmERS1*, and *GmETR2* have two duplicates and *GmEIN4* has four duplicates, whereas *GmERS2* exists as a single copy, like in *Arabidopsis*. It is clear that these ethylene receptors are not equally duplicated in soybean. Thus far, we do not know why these receptors have different numbers of duplicates. It is conceivable that the members of the first receptor family subgroup (ETR1 and ERS1) are more conservative because they play a more important role in ethylene signaling and plant responses to ethylene (Qu et al., 2007; Rivarola et al., 2009). Although we do not know why the members of the second subgroup (GmETR2, GmEIN4, and GmERS2) show such big differences in duplicate number, it is likely that alterations in the numbers of these receptors can increase the regulatory flexibility and complexity of plant responses to ethylene. Notably, our finding support the notion that soybean and *Medicago* also contain all types of ethylene receptors including ERS2, which is apparently not in agreement with the conclusion drawn by Gallie (2015a) that ERS2 homologs exist only in the *Brassicaceae*. The different conclusions about ERS2 are due to the incomplete data used in the previous study. Therefore, the conclusion drawn in the previous study need to be further analyzed using more completed data from various plant species. But our analysis results favor the hypothesis that ERS2 is the most newly evolved ethylene receptor because there is only a single copy of *ERS2*, which may arise after genome duplication (Gallie, 2015a). Interestingly, we found that *Medicago* contains a single copy of ETR1, ETR2, ERS1 and ERS2, but two copies of EIN4. It remains unknown whether extra copy (copies) of EIN4 in *Medicago* and soybean mediate the biological processes unique to legumes.

Interestingly, our domain analysis showed that all 11 receptors contain a GAF domain at their N-terminus, though some receptors have extra domains compared with their *Arabidopsis* homologs. Notably, the protein structures of ETR1 and ERS1 are highly conserved in *Arabidopsis* and soybean. Specifically, they share exactly the same domains [a GAF, a His kinase A (phospho-acceptor) domain, a His kinase domain, and a receiver domain], supporting the key role of ETR1 and ERS1 in ethylene perception and responses. It is worth noting that in GmETR2 and GmEIN4 half of the protein is similar to its *Arabidopsis* homolog, whereas the other half contains an extra His kinase A domain. Since the His kinase domain is responsible for dimerization with the phospho-acceptor domain, the evolution of the His kinase A domain suggests that these ethylene receptors have additional functions in mediating ethylene perception and the activation of downstream ethylene signaling events. However, the underlying molecular mechanism of these new ethylene receptor members is unknown. Further characterization of these proteins with an extra domain will help us understand how these receptors mediate ethylene perception and regulate plant responses to ethylene.

In *Arabidopsis*, five ethylene receptors also show tissue/organ or developmental stage specificity, although they are functionally redundant (Schaller and Bleecker, 1995; Hua and Meyerowitz, 1998; Guo and Ecker, 2004). In soybean, we found that the 11 ethylene receptors exhibit tissue/organ specificity. Some receptor genes have a similar expression pattern to their duplicates (e.g., *GmERS1a* and *GmERS1b*), which consistent with a low dn/ds ratio suggested the evolutionary conservation of these ethylene receptor genes. However, the duplicates of some receptor genes display a different expression pattern. For example, *GmEIN4a* was found to be highly expressed in leaf and nodule, whereas *GmEIN4b* showed its highest expression level in root. Notably, among these receptor genes, only *GmEIN4b* showed highest expression in root, whereas *GmERS2* showed the highest expression level in nodules. Furthermore, majority of the soybean receptor genes were found to be responsive to rhizobial infection. It worthy to note that eight of these ethylene receptor genes were downregulated by NF, indicating that these genes and the ethylene signaling pathway might play a role in the early process of nodulation in soybean. Interestingly, *GmERS1b* was highly induced by NF, suggesting a different role of this gene during rhizobia infection. Furthermore, we do not exclude the possibility that these ethylene receptor genes may also exhibit different expression patterns during nodule formation and organogenesis to mediate late development process of nodulation in soybean. These expression analysis results suggest that in addition to their differences in protein structure, these receptor genes are regulated at the transcriptional level. These multiple levels of regulation may enable the receptors to precisely and collaboratively modulate various biological processes in soybean. It is possible that apart from the general functions of ethylene receptors in higher plants, some members may be evolved to specifically regulate nodulation and SNF in soybean. Functional analysis of individual ethylene receptor genes will uncover the roles of these genes in plant development and symbiotic nodulation in soybean.

## Author Contributions

YW and XL conceived the study. YW and XL designed the experiments. WY, XW, CS, HW did the bioinformatics analysis. YW, JY, LZ, and ZS performed the experiments. YW and XL wrote the article.

## Conflict of Interest Statement

The authors declare that the research was conducted in the absence of any commercial or financial relationships that could be construed as a potential conflict of interest.

## Footnotes

1 https://phytozome.jgi.doe.gov/pz/portal.html

2 http://gsds2.cbi.pku.edu.cn

3 http://bioinformatics.psb.ugent.be/webtools/plantcare/html/

4 http://soybase.org/soyseq/

5 http://bar.utoronto.ca/efpsoybean/cgi-bin/efpWeb.cgi

6 http://gsds.cbi.pku.edu.cn/

7 http://plants.ensembl.org/index.html

8 http://pfam.xfam.org/search

9 http://www.cbs.dtu.dk/services/TMHMM-2.0/

10 http://bar.utoronto.ca/efpsoybean/cgi-bin/efpWeb.cgi

11 http://www.arabidopsis.org/

12 https://www.hiv.lanl.gov/content/sequence/SNAP/SNAP.html

## References

[B1] Abeles F. B. Morgan P. W. Saltveit M. E. Jr. (1992). *Ethylene in Plant Biology* 2nd Edn. New York, NY: Academic Press.

[B2] Bleecker A. B. Kende H. (2000). Ethylene: a gaseous signal molecule in plants. *Annu. Rev. Cell Dev. Biol.* 16 1–18. 10.1146/annurev.cellbio.16.1.1 11031228

[B3] Burg S. P. Burg E. A. (1967). Molecular requirement for the biological activity of ethylene. *Plant Physiol.* 42 144–152. 10.1104/pp.42.1.144 16656478 PMC1086501

[B4] Caba J. M. Recalde L. Ligero F. (1998). Nitrate-induced ethylene biosynthesis and the control of nodulation in alfalfa. *Plant Cell Environ.* 21 87–93. 10.1046/j.1365-3040.1998.00242.x

[B5] Cao W. H. Dong Y. Zhang J. S. Chen S. Y. (2003). Characterization of an ethylene receptor homolog gene from rice. *Sci. China C Life Sci.* 46 370–378. 10.1007/BF03192580 21072609

[B6] Chang C. Stadler R. (2001). Ethylene hormone receptor action in *Arabidopsis*. *Bioessays* 23 619–627. 10.1002/bies.1087 11462215

[B7] Chen Y. F. Randlett M. D. Findell J. L. Schaller G. E. (2002). Localization of the ethylene receptor ETR1 to the endoplasmic reticulum of *Arabidopsis*. *J. Biol. Chem.* 277 19861–19866. 10.1074/jbc.M201286200 11916973

[B8] Cho Y.-H. Yoo S.-D. (2015). Novel connections and gaps in ethylene signaling from the ER membrane to the nucleus. *Front. Plant Sci.* 5:733 10.3389/fpls.2014.00733 PMC4283510 25601870

[B9] Deng W. Wang Y. Liu Z. Cheng Z. L. Xue C. Y. (2014). HemI: a toolkit for illustrating heatmaps. *PLoS ONE* 9:e111988 10.1371/journal.pone.0111988 PMC4221433 25372567

[B10] Ferguson B. Lin M.-H. Gresshoff P. M. (2013). Regulation of legume nodulation by acidic growth conditions. *Plant Signal. Behav.* 8:e23426 10.4161/psb.23426 PMC3676511 23333963

[B11] Ferguson B. J. Indrasumunar A. Hayashi S. Lin M.-H. Lin Y.-H. Reid D. E. (2010). Molecular analysis of legume nodule development and autoregulation. *J. Integr. Plant Biol.* 52 61–76. 10.1111/j.1744-7909.2010.00899.x 20074141

[B12] Gallie D. R. (2015a). Appearance and elaboration of the ethylene receptor family during land plant evolution. *Plant Mol. Biol.* 87 521–539. 10.1007/s11103-015-0296-z 25682121

[B13] Gallie D. R. (2015b). Ethylene receptors in plants - why so much complexity? *F1000Prime Rep.* 7 39 10.12703/P7-39 PMC4479046 26171216

[B14] Gamalero E. Glick B. R. (2015). Bacterial modulation of plant ethylene levels. *Plant Physiol.* 169 13–22. 10.1104/pp.15.00284 25897004 PMC4577377

[B15] Goodlass G. Smith K. A. (1979). Effects of ethylene on root extension and nodulation of pea (*Pisum sativum* L.) and white clover (*Trifolium repens* L.). *Plant Soil* 51 387–395. 10.1007/BF02197785

[B16] Gresshoff P. M. Lohar D. Chan P.-K. Biswas B. Jiang Q. Reid D. (2009). Genetic analysis of ethylene regulation of legume nodulation. *Plant Signal. Behav.* 4 818–823. 10.4161/psb.4.9.9395 19847106 PMC2802810

[B17] Grobbelaar N. Clarke B. Hough M. C. (1971). The nodulation and nitrogen fixation of isolated roots of *Phaseolus vulgaris* L.III. The effect of carbon dioxide and ethylene. *Plant Soil* 35 215–223. 10.1016/j.syapm.2012.04.003

[B18] Guinel F. C. (2016). Ethylene, a hormone at the center-stage of nodulation. *Front. Plant Sci.* 6:1121 10.3389/fpls.2015.01121 PMC4714629 26834752

[B19] Guinel F. C. LaRue T. A. (1992). Ethylene inhibitors partly restore nodulation to pea mutant E 107 (*brz*). *Plant Physiol.* 99 515–518. 10.1104/pp.99.2.515 16668916 PMC1080493

[B20] Guo H. Ecker J. R. (2004). The ethylene signaling pathway: new insights. *Curr. Opin. Plant Biol.* 7 40–49. 10.1016/j.pbi.2003.11.011 14732440

[B21] Hua J. Meyerowitz E. M. (1998). Ethylene responses are negatively regulated by a receptor gene family in *Arabidopsis thaliana*. *Cell* 94 261–271. 10.1016/S0092-8674(00)81425-7 9695954

[B22] Hunter W. J. (1993). Ethylene production by root nodules and effect of ethylene on nodulation in *Glycine max*. *Appl. Environ. Microbiol.* 59 1947–1950. 16348969 10.1128/aem.59.6.1947-1950.1993 PMC182189

[B23] Jian B. Liu B. Bi Y. Hou W. Wu C. Han T. (2008). Validation of internal control for gene expression study in soybean by quantitative real-time PCR. *BMC Mol. Biol.* 9:59 10.1186/1471-2199-9-59 PMC2443375 18573215

[B24] Johnson P. R. Ecker J. R. (1998). The ethylene gas signal transduction pathway: a molecular perspective. *Annu. Rev. Genet.* 32 227–254. 10.1146/annurev.genet.32.1.227 9928480

[B25] Ju C. Yoon G. M. Shemansky J. M. Lin D. Y. Ying Z. I. Chang J. (2012). CTR1 phosphorylates the central regulator EIN2 to control ethylene hormone signaling from the ER membrane to the nucleus in *Arabidopsis*. *Proc. Natl. Acad. Sci. U.S.A.* 109 19486–19491. 10.1073/pnas.1214848109 23132950 PMC3511113

[B26] Kieber J. J. Rothenberg M. Roman G. Feldmann K. A. Ecker J. R. (1993). CTR1 a negative regulator of the ethylene response pathway in *Arabidopsis*, encodes a member of the raf family of protein kinases. *Cell* 72 427–441. 10.1016/0092-8674(93)90119-B 8431946

[B27] Larrainzar E. Riely B. K. Kim S. C. Carrasquilla-Garcia N. Yu H. J. Hwang H. J. (2015). Deep sequencing of the *Medicago truncatula* root transcriptome reveals a massive and early interaction between nod factor and ethylene signals. *Plant Physiol.* 169 233–265. 10.1104/pp.15.00350 26175514 PMC4577383

[B28] Lee K. H. Larue T. A. (1992). Exogenous ethylene inhibits nodulation of *Pisum sativum* L. cv Sparkle. *Plant Physiol.* 100 1759–1763. 10.1104/pp.100.4.1759 16653194 PMC1075861

[B29] Lee T. H. Tang H. Wang X. Paterson A. H. (2012). PGDD: a database of gene and genome duplication in plants. *Nucleic Acids Res.* 41 D1152–D1158. 10.1093/nar/gks1104 23180799 PMC3531184

[B30] Libault M. Farmer A. Joshi T. Takahashi K. Langley R. J. Franklin L. D. (2010). An integrated transcriptome atlas of the crop model *Glycine max*, and its use in comparative analyses in plants. *Plant J.* 63 86–99. 10.1111/j.1365-313X.2010.04222.x 20408999

[B31] Lohar D. Stiller J. Kam J. Stacey G. Gresshoff P. M. (2009). Ethylene insensitivity conferred by a mutated *Arabidopsis* ethylene receptor gene alters nodulation in transgenic *Lotus japonicus*. *Ann. Bot.* 104 277–285. 10.1093/aob/mcp132 19505874 PMC2710892

[B32] Miyata K. Kawaguchi M. Nakagawa T. (2013). Two distinct EIN2 genes cooperatively regulate ethylene signaling in *Lotus japonicus*. *Plant Cell Physiol.* 54 1469–1477. 10.1093/pcp/pct095 23825220

[B33] Nukui N. Ezura H. Minamisawa K. (2004). Transgenic *Lotus japonicus* with an ethylene receptor gene *Cm-ERS1/H70A* enhances formation of infection threads and nodule primordia. *Plant Cell Physiol.* 45 427–435. 10.1093/pcp/pch046 15111717

[B34] Nukui N. Ezura H. Yuhash K. I. Yasuta T. Minamisawa K. (2000). Effects of ethylene precursor and inhibitors for ethylene biosynthesis and perception on nodulation in *Lotus japonicus* and *Macroptilium atropurpureum*. *Plant Cell Phystol.* 41 893–897. 10.1093/pcp/pcd011 10965947

[B35] Oldroyd G. E. Downie J. A. (2008). Coordinating nodule morphogenesis with rhizobial infection in legumes. *Annu. Rev. Plant Biol.* 59 519–546. 10.1146/annurev.arplant.59.032607.092839 18444906

[B36] Penmetsa R. V. Cook D. R. (1997). A legume ethylene-insensitive mutant hyper infected by its rhizobial symbiont. *Science* 275 527–530. 10.1126/science.275.5299.527 8999796

[B37] Penmetsa R. V. Uribe P. Anderson J. Lichtenzveig J. Gish J.-C. Nam Y.-W. (2008). The *Medicago truncatula* of the *Arabidopsis EIN2* gene, sickle, is a negative regulator of symbiotic and pathogenic microbial interactions. *Plant J.* 55 580–595. 10.1111/j.1365-313X.2008.03531.x 18435823

[B38] Qu X. Hall B. P. Gao Z. Schaller G. E. (2007). A strong constitutive ethylene-response phenotype conferred on *Arabidopsis* plants containing null mutations in the ethylene receptors ETR1 and ERS1. *BMC Plant Biol.* 7:3 10.1186/1471-2229-7-3 PMC1781942 17224067

[B39] Rivarola M. McClellan C. A. Resnick J. S. Chang C. (2009). ETR1-specific mutations distinguish ETR1 from other *Arabidopsis* ethylene receptors as revealed by genetic interaction with RTE1. *Plant Physiol.* 150 547–551. 10.1104/pp.109.138461 19369589 PMC2689983

[B40] Schaller G. E. Bleecker A. B. (1995). Ethylene-binding sites generated in yeast expressing the *Arabidopsis* ETR1 gene. *Science* 270 1809–1811. 10.1126/science.270.5243.1809 8525372

[B41] Schlueter J. A. Lin J. Y. Schlueter S. D. Vasylenko-Sanders I. F. Deshpande S. Yi J. (2007). Gene duplication and paleopolyploidy in soybean and the implications for whole genome sequencing. *BMC Genomics* 8:330 10.1186/1471-2164-8-330 PMC2077340 17880721

[B42] Schmidt J. S. Harper J. E. Hoffman T. K. Bent A. F. (1999). Regulation of soybean nodulation independent of ethylene signaling. *Plant Physiol.* 119 951–959. 10.1104/pp.119.3.951 10069833 PMC32109

[B43] Severin A. J. Woody J. L. Bolon Y.-T. Joseph B. Diers B. W. Farmer A. D. (2010). RNA-seq atlas of *Glycine max*: a guide to the soybean transcriptome. *BMC Plant Biol.* 10:160 10.1186/1471-2229-10-160 PMC3017786 20687943

[B44] Shoemaker R. C. Schlueter J. Doyle J. J. (2006). Paleopolyploidy and gene duplication in soybean and other legumes. *Curr. Opin. Plant Biol.* 9 104–109. 10.1016/j.pbi.2006.01.007 16458041

[B45] Stacey G. Libault M. Brechenmacher L. Wan J. May G. D. (2006). Genetics and functional genomics of legume nodulation. *Curr. Opin. Plant Biol.* 9 110–121. 10.1016/j.pbi.2006.01.005 16458572

[B46] Suganuma N. Yamauchi H. Yamamoto K. (1995). Enhanced production of ethylene by soybean roots after inoculation with *Bradyrhizobium japonicum*. *Plant Sci.* 111 163–168. 10.1016/0168-9452(95)04239-Q

[B47] Tamura K. Nei M. Kumar S. (2004). Prospects for inferring very large phylogenies by using the neighbor-joining method. *Proc. Natl. Acad. Sci. U.S.A.* 101 11030–11035. 10.1073/pnas.0404206101 15258291 PMC491989

[B48] Tamura K. Peterson D. Peterson N. Stecher G. Nei M. Kumar S. (2011). MEGA5: molecular evolutionary genetics analysis using maximum likelihood, evolutionary distance, and maximum parsimony methods. *Mol. Biol. Evol.* 28 2731–2739. 10.1093/molbev/msr121 21546353 PMC3203626

[B49] Thompson J. D. Gibson T. J. Plewniak F. Jeanmougin F. Higgins D. G. (1997). The CLUSTAL_X windows interface: flexible strategies for multiple sequence alignment aided by quality analysis tools. *Nucleic Acids Res.* 25 4876–4882. 10.1093/nar/25.24.4876 9396791 PMC147148

[B50] Wang Y. Li P. Cao X. Wang X. Zhang A. Li X. (2009). Identification and expression analysis of miRNAs from nitrogen-fixing soybean nodules. *Biochem. Biophys. Res. Commun.* 378 799–803. 10.1016/j.bbrc.2008.11.140 19084500

[B51] Wang Y. N. Li K. X. Chen L. Zou Y. M. Liu H. P. Li D. X. (2015). MicroRNA167-directed regulation of the auxin response factors, GmARF8a and GmARF8b, is required for soybean (*Glycine max* L.) nodulation and lateral root development. *Plant Physiol.* 168 101–116. 10.1104/pp.15.00265 PMC4741323 25941314

[B52] Wang Y. N. Wang L. X. Zou Y. M. Chen L. Cai Z. M. Zhang S. L. (2014). Soybean miR172c targets the repressive AP2 transcription factor NNC1 to activate *ENOD40* expression and regulate nodule initiation. *Plant Cell* 26 4782–4801. 10.1105/tpc.114.131607 25549672 PMC4311200

