# Supplementary material for: Genome Wide Identification and Expression Profiling of Ethylene Receptor Genes during Soybean Nodulation
Source: Front Plant Sci. 2017 Jun 12;8:859. doi: 10.3389/fpls.2017.00859 (PMC5469071; doi:10.3389/fpls.2017.00859)
Supplement: Supplementary file 1 [file Table_1.docx]

**Supplementary Material**

**Table S1 | Information of primers used in this study.**

**Table S2 | The information about the ethylene receptors in *Arabidopsis* and soybean.**

**Table S3 | The digital expression data of soybean ethylene receptor genes in different tissues.**

**Table S4 | The digital expression data of ethylene receptor genes in different tissues of *Arabidopsis*.**

**Table S5 | Evolutional parameters for duplicated genes.**

**Figure S1 | The deduced amino acid sequences comparation of AtETR2, GmETR2a and GmETR2b.**

**Figure S2 | Association analysis between promoters and gene expression pattern.**

**Table S1 | Information of primers used in this study.**

| Primers Name | Forward Primer | Reverse Primer |
| --- | --- | --- |
| *GmETR1a-Real* | GCATTTAGGATGTGATGTAACAACTGC | CTTCGTAAACTTCTCATGTATACGGAC |
| *GmETR1b-Real* | GCATTTAGGATGTGATGTAACCACAGC | TTTAGTAAACTTTTCATGTATGCGGAT |
| *GmERS1a-Real* | GTCTTATGTGAACTCCTGCCG | CATTGGTTCATTAAAAAGCCG |
| *GmERS1b-Real* | GGTTTTATGCGAACTCCTGTTG | CAATACTGTCATTTTTATGAAC |
| *GmETR2a-Real* | GGATCCTTGTATATCGAGT | CTACTGTTGATCCCTATC |
| *GmETR2b-Real* | CAACGGGTGGACCTATGG | AAGGGTAATGGCGGTGGC |
| *GmEIN4a-Real* | CCAAGGTACTTGGATTTGG | GAAGAAAACAAAGAGAAG |
| *GmEIN4b-Real* | CACTTTATAACACAGATATAG | GGCTGAACCCAAGTACCTCA |
| *GmEIN4c-Real* | CTACTTAAATTGGGATTAG | CACATTACAATATAGATCC |
| *GmEIN4d-Real* | GAGTCTATAGTTAGTGGTGGC | CGTTGCAATATACTAGATCT |
| *GmERS2-Real* | AGAGGATAATCCAGTTGATGCAAGG | TGTCGGCCAACAAGACCTGC |

**Table S2 | The information about the ethylene receptors in *Arabidopsis* and soybean.**

**Table S3 | The digital expression data of soybean ethylene receptor genes in different tissues.**

The data was collected from http://bar.utoronto.ca/efpsoybean/cgi-bin/efpWeb.cgi and used for analyzing co-expression.

**Table S4 | The digital expression data of ethylene receptor genes in different tissues of *Arabidopsis*.**

The data was collected from http://www.arabidopsis.org/ and used for analyzing co-expression.

**Table S5 | Evolutional parameters for duplicated genes.**

The coding sequences collected from the Phytozome database were aligned with MEGA6 and parameters between paired genes were estimated with SNAP (<https://www.hiv.lanl.gov/content/sequence/SNAP/SNAP.html>). S: The number of potential synonymous substitutions (the average for the two compared sequences). N: The number of potential non-synonymous substitutions (the average for the two compared sequences). ps: The proportion of observed synonymous substitutions: Sd/S. pn: The proportion of observed synonymous substitutions: Sn/N. dn: The Jukes-Cantor correction for multiple hits of pn. ds: The Jukes-Cantor correction for multiple hits of ps. dn/ds: The ratio of non-synonymous to synonymous substitutions.

**Figure S1.
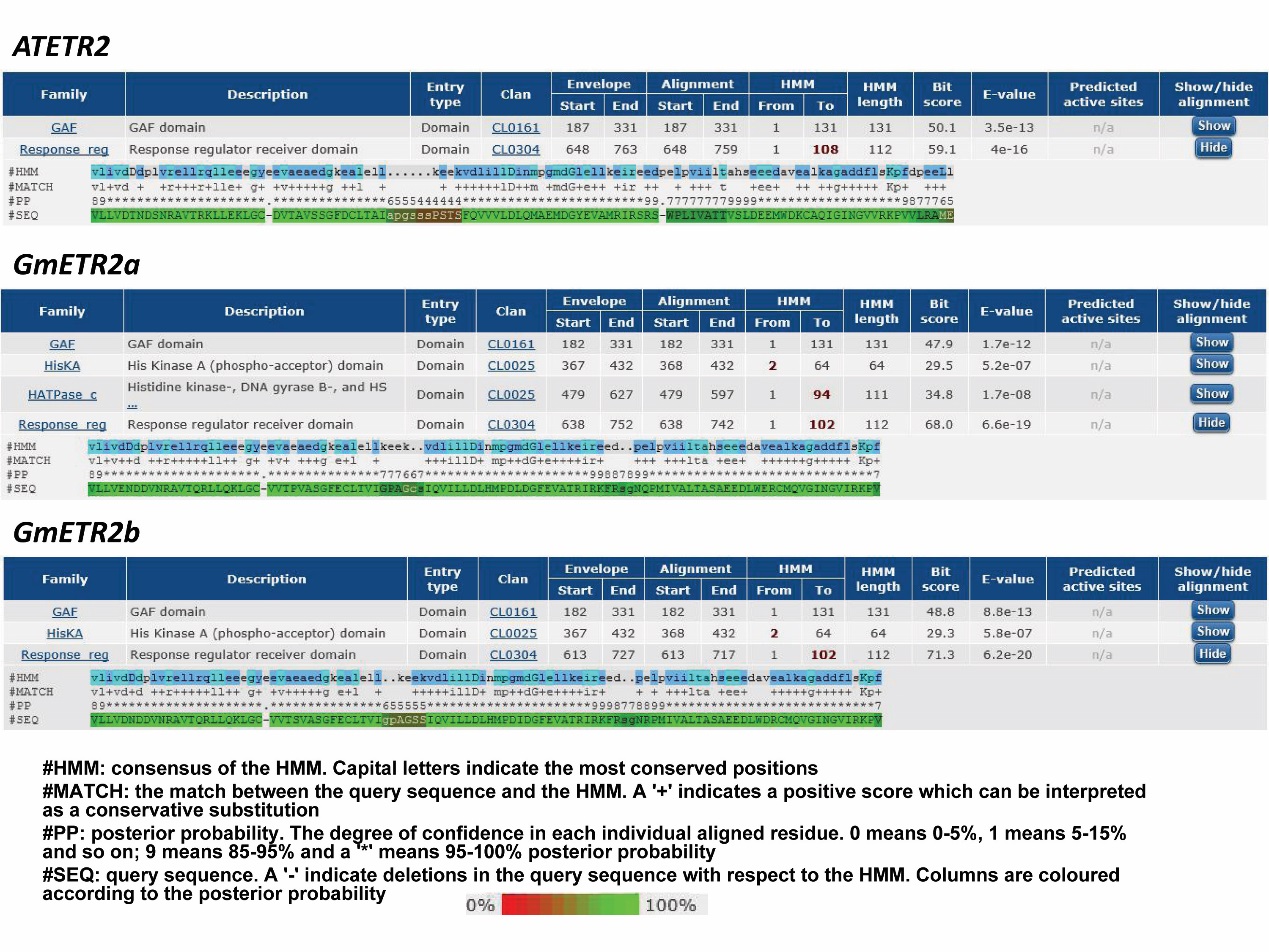
**

**Figure S1 | The deduced amino acid sequences comparation among** **AtETR2, GmETR2a and GmETR2b.** The deduced amino acid sequences collected from the Phytozome database were aligned and the conserved domain were analyzed using PFAM (http://pfam.xfam.org/).

**Figure S2.**


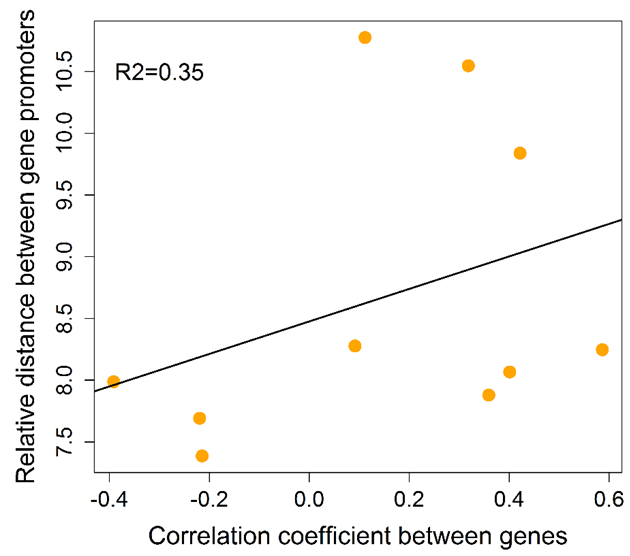

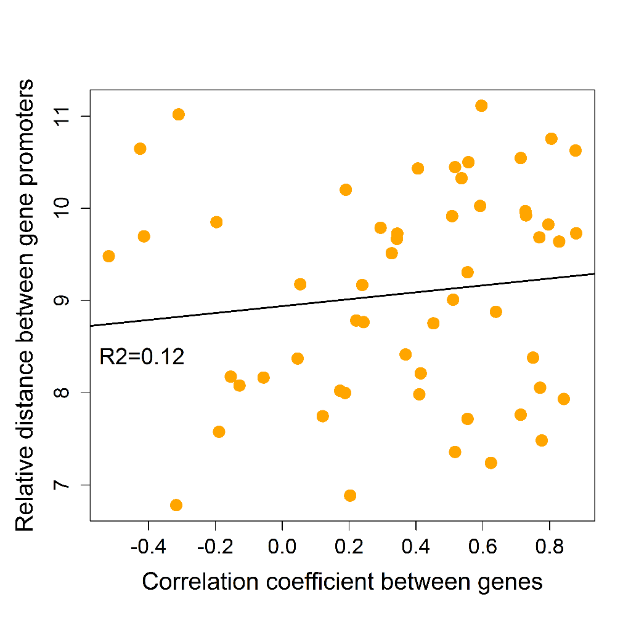


**A**

**B**

**Figure S2 | Association between the promoter sequence similarity and expressional pattern of ethylene receptor genes in soybean and *Arabidopsis*.** The expression data of each gene in 8 different tissues of soybean (<http://bar.utoronto.ca/efpsoybean/cgi-bin/efpWeb.cgi>) (A) and 47 tissues of *Arabidopsis* ethylene receptor genes collected from the website (<http://www.arabidopsis.org/>) (B) were used to be analyzed. The maximum composite likelihood distance between promoter sequence (2000 bp from TSS) were estimated with MEGA6. R2 indicates the relative correlation coefficient.
